# The transplant cohort of the German center for infection research (DZIF Tx-Cohort): study design and baseline characteristics

**DOI:** 10.1007/s10654-020-00715-3

**Published:** 2021-01-25

**Authors:** André Karch, Daniela Schindler, Andrea Kühn-Steven, Rainer Blaser, Klaus A. Kuhn, Lisa Sandmann, Claudia Sommerer, Markus Guba, Uwe Heemann, Jens Strohäker, Stephan Glöckner, Rafael Mikolajczyk, Dirk H. Busch, Thomas F. Schulz, for the Transplant Cohort of the German Center for Infection Research (DZIF Transplant Cohort) Consortium, Andreas Lehmann, Arnold Ganser, Berit Lange, Britta Maecker-Kolhoff, Burkhard Tönshoff, Christian Morath, Christina Rieger, Christine Falk, Christoph Schmaderer, Diana Pohle, Ekkehard Sturm, Elmar Jäckel, Florian Kohlmayer, Gabriele Anton, Gérard Krause, H.-Erich Wichmann, Heiko Mix, Jörg Janne Vehreschild, Julian Bucher, Juliane Hädicke-Jarboui, Karl-Heinz Weiss, Katrin Wagner, Lars Pape, Lorenz Frey, Lutz Renders, Mareike Verbeek, Mario Schiffer, Matthias Zirngibl, Michael M. Kreusser, Michael Neuenhahn, Michaela Geßner, Peter Lang, Silvio Nadalin, Stefan Meuer, Thomas Giese, Thomas Iftner, Thomas Illig, Tina Ganzenmüller, Tobias Welte, Wolfgang Bethge

**Affiliations:** 1 Institute of Epidemiology and Social Medicine, University of Münster, Münster, Germany; 2 German Center for Infection Research, Hannover-Braunschweig Site, Brunswick, Germany; 3 Department of Nephrology, Klinikum rechts der Isar of the Technical University Munich, Munich, Germany; 4 German Center for Infection Research, Munich Site, Munich, Germany; 5 German Research Center for Environmental Health, Helmholtz Zentrum München, Munich, Germany; 6 Institute of Medical Informatics, Statistics and Epidemiology, Technical University Munich, Munich, Germany; 7 Epidemiology, Helmholtz Center for Infection Research Braunschweig, Brunswick, Germany; 8 Institute for Medical Epidemiology, Biometry and Informatics, Medical Faculty, Martin-Luther University Halle-Wittenberg, Halle, Germany; 9 Institute for Medical Microbiology, Immunology and Hygiene (MIH), Technical University of Munich, Munich, Germany; 10 Institute of Virology, Hannover Medical School (MHH), Hannover, Germany; 11 German Center for Infection Research, Heidelberg Site, Heidelberg, Germany; 12 Nierenzentrum Heidelberg, Heidelberg, Germany; 13 Department of General, Visceral and Transplantation Surgery, University Hospital, LMU Munich, Munich, Germany; 14 German Center for Infection Research, Tübingen Site, Tübingen, Germany; 15 University Hospital for General, Visceral and Transplant Surgery, Tübingen, Germany; 16 Department of Gastroenterology, Hepatology and Endocrinology, Hannover Medical School (MHH), Hannover, Germany

**Keywords:** Clinical cohort study, Organ transplantation, Immunosuppression, Infection

## Abstract

Infectious complications are the major cause of morbidity and mortality after solid organ and stem cell transplantation. To better understand host and environmental factors associated with an increased risk of infection as well as the effect of infections on function and survival of transplanted organs, we established the DZIF Transplant Cohort, a multicentre prospective cohort study within the organizational structure of the German Center for Infection Research. At time of transplantation, heart-, kidney-, lung-, liver-, pancreas- and hematopoetic stem cell- transplanted patients are enrolled into the study. Follow-up visits are scheduled at 3, 6, 9, 12 months after transplantation, and annually thereafter; extracurricular visits are conducted in case of infectious complications. Comprehensive standard operating procedures, web-based data collection and monitoring tools as well as a state of the art biobanking concept for blood, purified PBMCs, urine, and faeces samples ensure high quality of data and biosample collection. By collecting detailed information on immunosuppressive medication, infectious complications, type of infectious agent and therapy, as well as by providing corresponding biosamples, the cohort will establish the foundation for a broad spectrum of studies in the field of infectious diseases and transplant medicine. By January 2020, baseline data and biosamples of about 1400 patients have been collected. We plan to recruit 3500 patients by 2023, and continue follow-up visits and the documentation of infectious events at least until 2025. Information about the DZIF Transplant Cohort is available at https://www.dzif.de/en/working-group/transplant-cohort.

**Supplementary information:**

The online version of this article (10.1007/s10654-020-00715-3) contains supplementary material, which is available to authorized users.

## Background

An aging population and the constantly growing prevalence of chronic diseases in high-income countries lead to an increased number of individuals in need of solid organ or stem cell transplantation. Infections in transplant recipients have a decisive impact on graft function and survival of the transplant recipient [1–4]. In both, kidney and liver transplant recipients, infection is the leading cause of death in the immediate post-transplant period [1–4]. In patients after lung transplantation, non-CMV infections account for about 40% of deaths occurring during the first 30 days after transplantation, and are responsible for 20% of deaths after the first year following transplantation [5]. In addition, infections in transplant recipients are responsible for an increased loss of transplanted organs [6], the development of malignant diseases such as EBV-associated transplant lymphoma [7], non-melanoma skin cancer due to cutaneous human papillomavirus infections [8], and transplant-associated Kaposi Sarcoma caused by KSHV/HHV8 [9], as well as a reduced quality of life, and increased health care costs [6].

Despite their important role for the prognosis of transplant recipients, there are still many open questions with respect to the prevention, early detection, therapy, and consequences of post-transplant infections. For example, little is known about the long-term consequences of many infections on graft survival/function and graft-versus-host disease (GvHD), the role of individual susceptibility to bacterial, viral, and fungal colonisation under immunosuppression, the evolution of the antiviral T cell repertoire, the long-term impact of antiviral therapy on graft and patient survival, or changes in the physiological microbiome or virome that may have a bearing on colonisation with pathogenic microbes. The viral aetiology of the most frequent tumour in transplant recipients, non-melanoma skin cancer (NMSC), remains controversial, and a possible viral aetiology of other malignancies occurring at increased incidence in transplant recipients seems plausible. Prospective cohort studies allow to link infection with, and the immune response to particular viruses, as determined by virus detection, viral load, antibody reactivity, virus-specific T-cells, to the development of these transplant complications. Although there are many established cohort studies and disease registries in the field of transplant medicine ([10, 12–17]; Supplementary Table 1), most of them do not allow a detailed assessment of infections due to a lack of biosamples or data about infectious outcomes. One exception is the Swiss Transplant Cohort Study (STCS) [10], which includes a detailed assessment of infectious diseases at baseline and during follow up. We developed our cohort to be compatible with the STCS to allow joint analyses in the future. The acquisition of medical data and the collection of biosamples, not only at fixed times, but also in the case of infectious events, represents a particular strength of the DZIF transplant cohort. By integrating several clinical centres at the German Center for Infection Research partner sites (Hannover Medical School, Heidelberg University Hospital, University Hospital Munich rechts der Isar, LMU Klinikum Munich, University Hospital Tübingen), large numbers of patients can be recruited, and biosamples can be collected.

To study the impact of rare infections on transplant function and survival, a large sample size is needed. More than 3500 solid organ transplantations and 7000 stem cell transplantations are performed in Germany every year [11], and several transplant centres are located at partner institutions of the German Center for Infection Research. With the help of these transplant centres, we initiated a prospective cohort of transplant recipients using systematic criteria for enrolment, data collection and sample collection. Information about the DZIF Transplant Cohort is available at https://www.dzif.de/en/working-group/transplant-cohort.

## Study design and methodology

### Study population and recruitment

The DZIF Transplant Cohort (DZIF Tx-Cohort) is designed as a multicentre prospective cohort study within the organizational structure of the German Center for Infection Research (Deutsches Zentrum für Infektionsforschung; DZIF). It currently enrols patients from five of the largest German university transplant centres, and collects clinical information as well as biological samples of donors and transplanted patients. The five centres together cover between 20 (kidney) and 70 (lung) percent of all solid organ transplants in Germany. While it can be assumed that liver, lung and heart transplant patients treated in the five centres are representative for Germany, this is less clear for kidney and stem cell transplant patients, because the proportion of patients treated outside the five centres is considerably higher. Within the DTIF Tx-Cohort, transplant recipients are seen at regular follow-up visits and ad hoc, when infectious complications occur. Inclusion criteria are restricted to being listed for a transplantation of heart, lung, liver, kidney, pancreas, or stem cells. An inclusion in the study is only possible, if written informed consent is given by the patient or his/her legal guardian. For paediatric patients, a specific age-dependent consent process was developed together with the responsible ethics committees. The informed consent process had been audited by the local ethics committees, and by the data protection officers of all participating hospitals. Of those patients approached for informed consent, 94% could be included in the study (ranging from 47% for heart transplants to 97% for kidney transplants).

### Data collection and baseline examination

After recruitment, individual data collection starts with a baseline visit (at time of transplantation). Follow-up visits are performed in line with the regular follow-up schedule of the transplant centre (0, 3, 6, 9, 12 months, and yearly thereafter). If the transplant patient visits the centre outside the planned follow-up schedule (e.g. for infectious complications), an additional visit should be included in the database. Follow-up will be continued as long as the patient is seen at the centre. All follow-up visits are entered in the database by trained study nurses using the DZIF Tx-Cohort Standard Operating Procedures (SOPs).

**Fig. 1:**
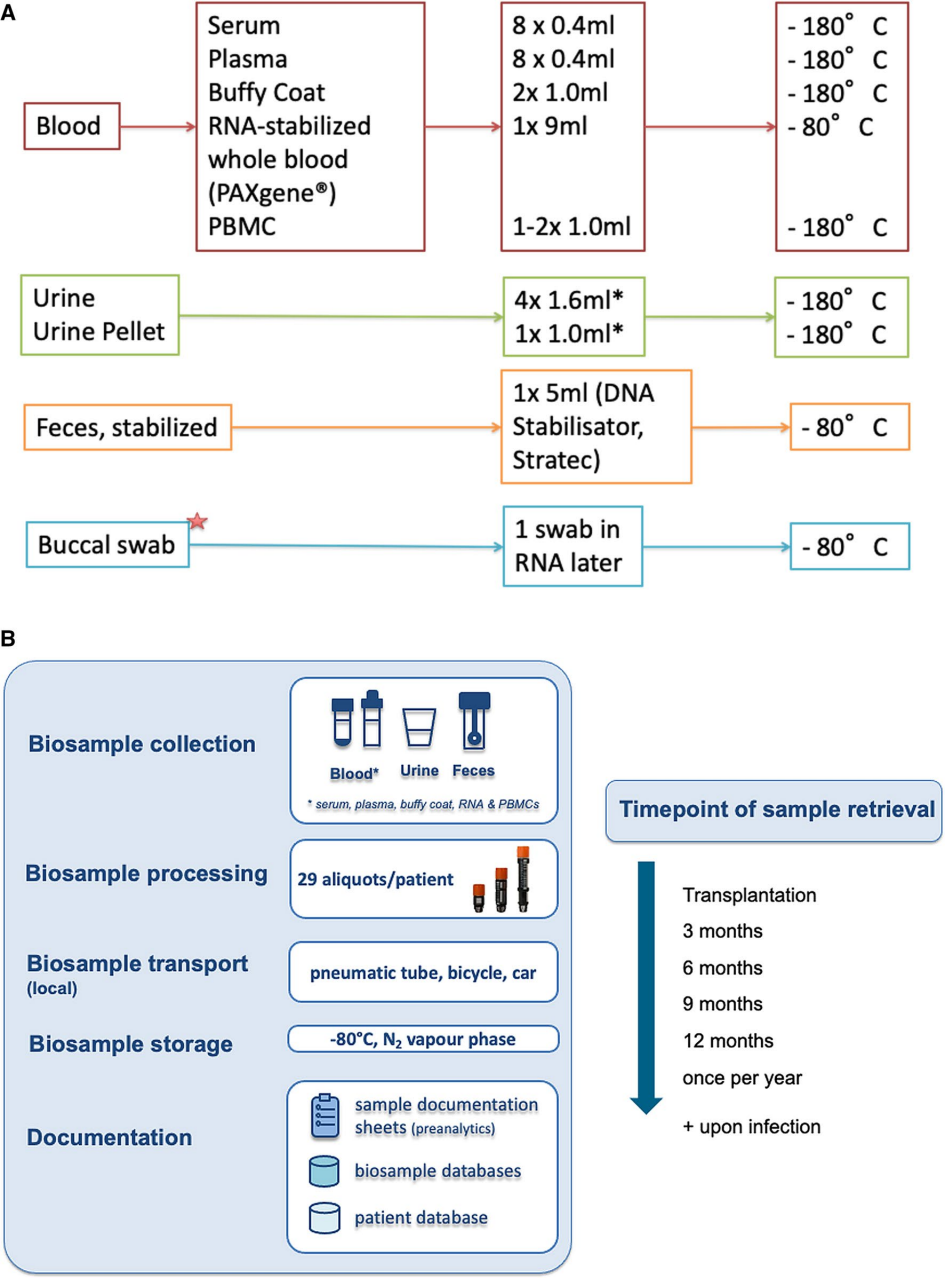
Sampling of biomaterials (**a**), processing and storage strategy (**b**) in the DZIF transplant cohort. *Buccal swabs are not performed at every center

Collected data are entered into a web-based electronic case report form (eCRF). For each visit, a mandatory minimal dataset needs to be collected (Fig. 1). For the baseline visit, this minimal dataset includes general baseline information for recipient and donor (including results of genetic and serologic tests at time of transplantation as well as laboratory parameters), on the process of transplantation itself (including conditioning treatments, intra- and post-operative parameters), and potential post-transplant anti-infective prophylaxis.

Additional data are entered dependent on the transplanted organ and potential complications according to pre-defined standard operating procedures described in a central data entry manual. Biosamples are obtained by each center and stored in cooperation with local established biobanks. Since there is a centralized biobanking platform in the German Center for Infection Research, namely the central biosample registry (DZIF-ZBR) located at the Helmholtz Zentrum München, metadata of locally stored biosamples are also available centrally. Transfer of data from local databases (e.g. Laboratory Information Management Systems for biosample data) and the central cohort database occurs on a regular basis (for patient/medical data) via a web-based interface into the DZIF-ZBR.

### Organization of follow-up visits

Follow-up visits include a retrospective documentation of patient status and relevant events that occurred between the previous regular and the current visit. Apart from the mandatory minimum dataset collected at each routine follow-up visit (consisting of the survival status of the patient, the function of the transplanted organ, laboratory parameters and immunosuppressive medication), additional data on complications like infections (if not already documented as an extracurricular visit) or rejection are documented.

To ensure high adherence with respect to both data and biosample collection during follow-up, several process optimization measures have been implemented. Patients receive an identification (ID) card with an overview of their follow-up appointments at time of enrolment. Contact phone numbers of study nurses are printed on the ID card together with the request, that patients and involved medical staff inform the responsible study nurse in case of any unscheduled visit. This is particularly relevant if patients are treated for an infectious event or other complications in hospitals other than the recruiting transplant centres. To alert healthcare workers involved in the transplant cohort when a patient attends the hospital outside the regularly scheduled follow-up visits because of acute disease, alert message systems were implemented within the hospital information systems of the participating centres. Every time, a study patient is seen as an inpatient or outpatient, the responsible study nurse is informed by the system.

### Linkage to secondary data

One major limitation for long-term cohort studies on solid organ and stem cell transplantation in the German healthcare system is that transplanted patients (dependent on the organ) are under the care of resident physicians or other hospitals closer to their home location in the years following transplantation. To be able to collect a minimum dataset from these patients when regular visits at the transplant centres have come to an end, collaborations with health insurance companies are currently being established so that data from electronic health records can be linked to the DZIF Tx-Cohort. By doing so, general information regarding vital status (dead or alive) of the patient would be available, as well as transplant-specific information about medication, outpatient diagnoses and hospital visits. This would add substantial knowledge about the individual infection history of each transplant recipient. Moreover, we would be able to obtain information about health of the patients beyond the issues of transplantation medicine, including treatment in other hospitals and in the outpatient sector.

### Sample size and power estimations

One strength of this project is the close collaboration across different transplant centres and the participating medical and surgical disciplines. By bringing together solid organ and stem cell transplantations at five of the largest transplant centres in Germany, this study will be able to combine extensive phenotyping and biosample collections with a sample size that provides us with as sufficient statistical power to perform in-depth analysis for rare infectious complications (Supplementary Table 2). Over a period of 10 years, about 3500 patients are expected to be available for further analyses.

## Study organisation

### Central cohort database

The central cohort database is based on the open source system DIS (Data Integration System), developed mainly out of the Leading Edge Cluster m4 [18]. DIS provides a secure identity management component and functionality for the management of observational data and biosamples. Important features are the ability of the system to integrate data from various sources and state-of-the-art security features, including two-tier pseudonymisation, encryption of data-at-rest, and data-in-transit as well as role-based access and audit trails. Its underlying ethics and data protection concept has been approved by the relevant institutional review boards, and it has been reviewed by the data protection commissioner of Bavaria [19].

Following the specifications of the data protection concept, a central DIS instance was set up at the Technical University of Munich Medical Center. Forms for web-based data entry were created according to the study protocol, including all relevant clinical data items for baseline examination and follow-up. Information on sample collection and the allocation of samples to patients are also recorded in the central database, while further sample handling and tracking is managed locally in the biobank system of the respective centre. A data entry manual was developed and range checks and other plausibility checks within the entry forms were defined and implemented to provide the data collectors with rapid feedback on possible incorrect entries to increase data quality. Regular data exports from the central cohort database are used for further quality management measures (see section on quality management for clinical data).

The user administration of the central database was configured based on the data protection concept of the DZIF Tx-Cohort and roles based thereon (see section on ethics and data protection). In each centre, an information technology (IT) manager was appointed who is responsible for the local user administration and the instruction of the users and who represents the primary link to the central IT and quality assurance managers.

Before the start of the productive phase, the central database was intensively tested, including two pilot phases with real data (see respective chapter below), and embedded in the real processes of the participating clinics. Experiences from the pilot phases and analysis results on data quality were used to adapt and optimize the central database.

### Collection and storage of biosamples

Planning of collection, storage and documentation of biosamples was conducted with support of the Infrastructure Biobanking in the German Center for Infection Research. Based on the mandate and the aim of the cohort, biomaterials were defined and costs concerning collection material, staff, IT, and storage were calculated on the basis of experiences of HMGU Biobank (Munich). Minimal standards were developed taking into account the individual logistical circumstances and infrastructure at each involved centre. These standards defined the maximum allowed deviations to guarantee a comparable sample quality at the five different partner institutions. Additionally, ID management (coordinated at HMGU Munich) as well as the documentation of pre-analytical items in sample documentation sheets was developed under support of the Infrastructure Biobanking. The sample documentation sheets include the documentation of all relevant time stamps (time of retrieval, processing, storage) and deviations from standards defined in the respective SOPs. Documentation is performed in local laboratory information and management systems (LIMS). Biosamples are stored at the local partner sites, in partner biobanks according to central SOPs (Fig. 1).

### Quality management for clinical data

The DZIF Tx-Cohort applies continuous data quality assurance procedures to the collected clinical data at baseline and during follow up. The evaluation of data quality uses standards developed within the consortium based on data quality standards for epidemiological cohort studies [20]. All applied quality assurance procedures aim to ensure that study performance, data collection, data entry as well as integrity of captured patient-related information are in agreement with Good Clinical Practice regulations.

Previous studies have shown that the overall quality of clinical databases needs to be assessed continuously [21]. While it is possible to improve data quality at data entry by validation via e.g. pulldown menus or radio buttons (which are implemented in the DZIF Tx-Cohort database), there is also a need for clinicians and study nurses to check and correct erroneously completed forms. To ensure a high standard of data quality, a framework for ongoing data quality evaluation that continuously assesses data completeness, data correctness and data timeliness [22] has been implemented in the DZIF Tx-Cohort using an internal database feedback system. Results of the data quality assessment are reported in the form of dashboards. These dashboards provide individual feedback for study nurses, (local) investigators and the cohort principal investigators. Study nurses are provided with case-based feedback and recommendations on how to improve data completeness. Investigators are provided with study performance measures (e.g. recruitment rates, follow-up completeness, overview of biosamples) to ensure consistent data processes within the local study centres. Furthermore, gamified features are implemented in the dashboard, showing leaderboards grouped by centres with an overview of overall data quality. Applying gamification within the clinical context provides the possibility to motivate users to enter more and correct information into the eCRFs. Feedback is sent to all study personnel monthly, but it is also implemented within the data management to provide real-time feedback for project managers, investigators and study nurses.

### Quality management for biosamples

**Fig. 2:**
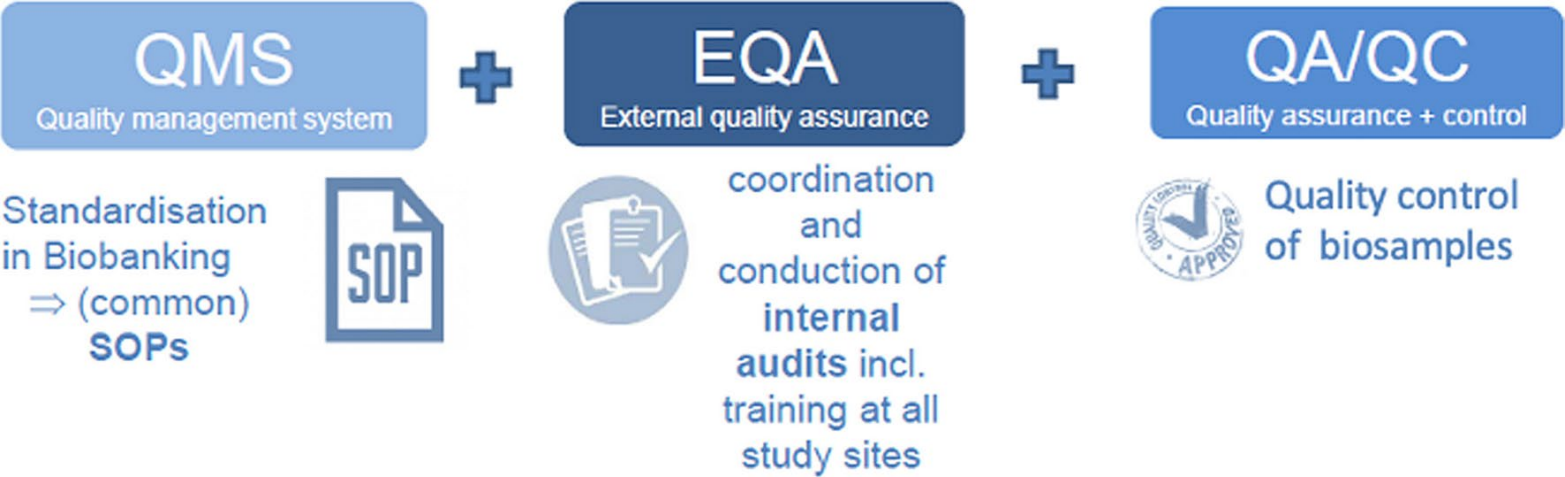
Quality management strategy for the biosampling module in the DZIF transplant cohort

A three-step quality management system for biosample collection was developed. First, minimal standards for sample retrieval, processing, documentation and storage were developed and agreed on between the partner sites. After a phase of adoption and usage, the minimal standards, documentation sheets, and instructions (for patients/staff) were combined in a biomaterial handbook. Second, internal audits (friendly audits) were conducted at the partner sites to monitor possible deviations from the biomaterial handbook. Audits were planned, prepared, and conducted by the Infrastructure Biobanking, and audit reports were written and handed out to the partner sites, the management board, and the principal investigators. Content of the audits are the identification of deviations to standards in the biosample process chain “from needle to freezer” including documentation. Annual sequel audits are scheduled. Third, the quality of currently collected biosamples (plasma), randomly chosen from patients at each partner site, is controlled with special inclusion criteria by using state-of-the art methods like metabolomics and miRNA biomarker analyses. Altogether, the quality management measures complement each other to prevent pre-analytical mistakes, detect handling errors and to show the fitness-for-purpose of the collected biomaterials. Moreover, harmonization between the partner sites and the correction of deviations lead to a high and comparable biosample quality for reproducible and reliable analyses (Fig. 2).

### Pilot studies

Two pilot studies were conducted with a sample size of n = 14 (first pilot study) and n = 98 (second pilot study) to test the feasibility and functionality of the defined procedures. The first pilot study aimed mainly at testing the feasibility of the developed concept in a routine care setting. Durations of study visits and data entry were collected and analysed; SOPs for biosample management were tested at the different centres. Pilot study 1 was analysed quantitatively (particularly with respect to duration of processes, and quality of data) as well as qualitatively in focus groups. All processes, data collection tools, SOPs, and eCRFs were adjusted according to the results of pilot study 1.

The aim of the second pilot study was to test the final recruitment strategy, the data and biosample collection tools as well as the SOPs in a real-life setting across all partner sites and all types of transplanted organs. Again, analyses were performed both quantitatively and qualitatively. Moreover, recruitment rates during pilot study 2 were used to estimate sample sizes during the main phase of the study.

Several items in the eCRFs were changed, removed, added, or corrected based on the results of pilot study 2. The data entry manual provided for pilot phase 2 was updated so that data entry problems could be minimized.

### Ethics approval and data protection

The ethics concept of the DZIF Tx-Cohort was developed in close collaboration with all ethics committees at the participating centres. Ethics approval was granted by all Ethics Committees (Hannover Medical School Nr 6534, Medical Faculty of the University of Heidelberg Nr S-585/2013, Medical Faculty of the TU Munich Nr 5926/13, LMU Munich Nr 380-15, University Hospital Tübingen Nr 327/2014BO1).

The data protection concept (building on the m4 data security standards) was approved by all local data protection officers (Hannover Medical School 25 August 2014, University Hospital Heidelberg 10 September 2014, LMU Munich 09 September 2015, TU Munich 01 October 2013, University Hospital Tübingen 18 August 2014). All documents were adopted according to the new Data Protection Regulations (GDPR) in May 2018.

## Baseline characteristics of the first 1.389 study participants

After 18 months of the main phase of the DZIF Tx-Cohort, 1.389 study participants were enrolled. The majority of patients received a kidney transplantation (59.0%). Up to this point, no lung transplant patients were included. Some baseline characteristics of the study participants are shown in Table 1.

**Table 1:** Baseline characteristics and available aliquots of biosamples collected at baseline of study participants of the DZIF Tx-Cohort study enrolled before January 2020

Characteristics	All (n = 1.389)	Transplanted organ				
		Heart (n = 18)	Liver (n = 294)	Kidney (n = 820)	Pancreas (n = 31)	Stem cells (n = 226)
Age (median, range)	50	56 (35–66)	56 (2–74)	54 (2–82)	42 (26–70)	50 (4–79)
Female sex (%)	472 (33.9%)	5 (27.8%)	102 (34.7%)	274 (33.4%)	9 (29.0%)	82 (36.3%)
Recipients of organs from living donors (%)	491 (35.3%)	0 (0%)	12 (4.0%)	253 (63.8%)	0 (0%)	226 (100%)
Serum aliquots available (n =)	14.450	170	3.325	8.019	303	2.633
EDTA aliquots available (n =)	16.697	177	4.150	8.990	353	3.027
PBMC aliquots (n =)	1.654	0	413	621	13	607
Isolated RNA aliquots (n =)	2.984	23	614	1.749	78	520
Faeces aliquots available (n =)	835	0	182	390	14	249
Urine aliquots available (n =)	9.463	60	1.571	6.204	237	1.391

## Funding, governance, and management

### Funding

Open Access funding enabled and organized by Projekt DEAL. The DZIF Tx-Cohort is funded by the German Ministry of Education and Research via the German Center for Infection Research (DZIF, Funding Number TTU 07.701). It is funded as an infrastructure in the DZIF Translational Thematic Unit “Infections of the Immunocompromised host”. Each participating centre receives funds for personnel and/or consumables, based on the number of recruited patients. Funding is linked to regular DZIF funding periods, and will be reassessed for every new period.

### Governance

The DZIF Tx-Cohort is organized as an independent incorporated society linked directly to the German Center for Infection Research. Details about the governance structure can be found at https://www.dzif.de/en/working-group/transplant-cohort.

### Rights to access

Data and biosamples collected in the DZIF Transplant Cohort are generally available to the scientific public following a pre-defined application process.

The application consists of three parts: Preliminary application, full application and—depending on review and decision—transfer of applied data and/or samples.

A detailed description of the process together with the respective forms for each step is available at https://www.dzif.de/en/working-group/transplant-cohort.

## Conclusion

The DZIF Tx-Cohort offers a unique platform for research on infections in transplant recipients. By combining state of the art biosample collection and storage with a high level of phenotypic and clinical information, many unsolved research questions can be tackled for the first time. Examples of the issues to be addressed are the identification of biomarkers that predict the individual risk for a clinically severe infection with one of the typical opportunistic pathogens encountered in transplant recipients, examination of changes in the gut microbiome in transplant recipients together with possible metabolic processes in the blood and, in the long term, whole genome- and epigenome-based approaches for the identification of cellular biomarkers predicting graft survival/development of acute and/or chronic GvHD, susceptibility to infection, or adverse clinical outcome after infection. Data collected in the cohort will also allow the evaluation of the long-term effects of antibacterial and antiviral treatment on graft outcome. Questions regarding the risk for and outcome of infections in transplant recipients and their impact on organ function and survival are among those to be answered with the help of the DZIF Tx-cohort. A state-of-the-art quality management concept as established in the cohort is necessary to provide data and biosamples suitable to tackle these important research questions.

## Electronic supplementary material

Below is the link to the electronic supplementary material.

Supplementary material 1 (DOCX 13 kb)
