# Supplementary material for: The transplant cohort of the German center for infection research (DZIF Tx-Cohort): study design and baseline characteristics
Source: Eur J Epidemiol. 2021 Jan 25;36(2):233–41. doi: 10.1007/s10654-020-00715-3 (PMC7987595; doi:10.1007/s10654-020-00715-3)
Supplement: Supplementary file 1 — Supplementary material 1 (DOCX 13 kb) [file 10654_2020_715_MOESM1_ESM.docx]

Supplementary Table 1: Selected cohort studies and registries in the field of transplant medicine

| **Cohort name** | **Start of cohort** | **Covered organs** | **Study population** | **Sample size (most recent information)** | **Length of follow up** | **Detailed information about infections** | **Biosamples** |
| --- | --- | --- | --- | --- | --- | --- | --- |
| STCS (12) | 2008 | All solid | All Swiss Tx centers | 4,565 | Heterogeneous (median 3.7 years) | Yes | Plasma, DNA, viable cells |
| CTS Heidelberg (13) | 1982 | All solid | 400 centers in 45 countries | 700,000 | Heterogeneous  (92% with more than 10 years) | No | No |
| OPTN (14) | 1987 | All solid | Population-based in the US | 774,198 | Heterogeneous  (up to several years) | No | No |
| A2ALL (15) | 1998 | Liver (living Tx only) | 9 US centers | 2,182 | Heterogeneous  (up to several years) | Basic information available | Plasma, serum, DNA, biopsy (not for all cases) |
| ISHLT (16) | 1980 | Lung, heart | >290 centers worldwide | 213,340 | Heterogeneous  (up to several years) | Basic information available | No |
| ELTR (17) | 1985 | Liver | 137 centers in Europe | 147,161 | Heterogeneous  (up to several years) | No | No |
| ANZDATA (18) | 1978 | All solid | Population-based in Ozeania | 11,687 | Annual active update of key information | No | No |

Supplementary Table 2: Estimated power to detect pre-defined effect sizes (measured as Hazard Ratios) based on different sample sizes

|  | **Hazard Ratio (HR)** | | | |
| --- | --- | --- | --- | --- |
| **Sample size** | 1.2 | 1.5 | 1.8 | 2.0 |
| **200** | 0.09 | 0.30 | 0.55 | 0.69 |
| **400** | 0.15 | 0.53 | 0.84 | 0.94 |
| **800** | 0.25 | 0.82 | 0.99 | >0.99 |
| **1.200** | 0.35 | 0.94 | >0.99 | >0.99 |
| **1.600** | 0.45 | 0.98 | >0.99 | >0.99 |
| **2.000** | 0.55 | >0.99 | >0.99 | >0.99 |
| **3.000** | 0.70 | >0.99 | >0.99 | >0.99 |
